# Epidemiologic trends of cleft lip and/or palate in Switzerland

**DOI:** 10.1186/s12903-025-05500-w

**Published:** 2025-01-22

**Authors:** Joël Beyeler, Anic Lauener, Christos Katsaros, Giorgio C. La Scala, Martin Degen

**Affiliations:** 1 Department of Orthodontics and Dentofacial Orthopedics, University of Bern, Bern, Switzerland; 2 Department of Restorative, Preventive and Pediatric Dentistry, School of Dental Medicine, University of Bern, Bern, Switzerland; 3 Laboratory for Oral Molecular Biology, Department of Orthodontics and Dentofacial Orthopedics, University of Bern, Freiburgstrasse 3, Bern, 3010 Switzerland; 4 Pediatric Plastic Surgery, Division of Pediatric Surgery, Department of Pediatrics, University Hospital of Geneva, Geneva, Switzerland

**Keywords:** Cleft lip, Cleft palate, Craniofacial abnormalities, Orofacial cleft, Prevalence, Epidemiology, Switzerland

## Abstract

**Background:**

Epidemiologic data on the number of cleft lip and/or palate (orofacial cleft (OFC)) births in Switzerland are currently sparse. However, this knowledge is essential for better understanding the etiologies underlying the various cleft phenotypes and providing expectant parents with the best possible healthcare planning and counseling.

**Methods:**

This is the first descriptive study to report data on the prevalence of the various cleft types, their sex, and regional distributions in Switzerland. Data for the years 1998–2021 were obtained from the Swiss Federal Statistical Office. Due to the notable initial underreporting of cleft cases from 1998 to 2006, this period was omitted from the final analyses.

**Results:**

Between 2007 and 2021, the prevalence of all Swiss OFC cases per 10,000 live births was 12.5, with a stable trend. Cleft lip was the least common anomaly. Except for cleft palate, which was more common in females, males were generally more affected by OFC than females. There was no discernible regional trend for any of the malformations, even though the prevalence differed throughout the seven Swiss regions.

**Conclusions:**

This study presents the first descriptive epidemiologic profiles for OFCs in Switzerland and emphasizes the importance of nationwide OFC registries with an accurate and reliable reporting system for the benefit of current and future patients with clefts, their parents or caregivers, and society as a whole.

**Supplementary Information:**

The online version contains supplementary material available at 10.1186/s12903-025-05500-w.

## Background

Despite being among the most prevalent congenital craniofacial defects, orofacial clefts (OFCs) still cause serious psychosocial and emotional difficulties for both the affected individuals and their families [1, 2]. It is considered that roughly 1:700 newborns are affected by this heterogenous malformation worldwide [3, 4], but the frequency of cases varies greatly depending on geography and socioeconomic status [5–7]. While about 70% of OFC cases are non-syndromic, the remaining 30% do exhibit additional anomalies, making them syndromic [8]. In the most basic classification system, based on embryological and epidemiological differences, OFCs can be divided into two categories: cleft lip without (CL) or with cleft palate (CLP), and cleft palate (CP) [9–11]. While disturbances in the morphogenesis of the lip and/or primary palate might cause CL or CLP, failures to complete the formation of the subsequently developed secondary palate might result in CP [12, 13].

OFCs have a highly complex and multifaceted etiology, involving the interplay of genetic, behavioral, and environmental factors [4, 7, 14, 15]. Therefore, identifying the modifiable and non-modifiable OFC risk variables poses a serious challenge for potential future health interventions, preventive measures, and parent counseling. To achieve these objectives, national OFC registries are critical because they allow for the ongoing identification of individuals affected by OFC, including their geographic location and potential environmental exposures.

The “European Registration of Congenital Anomalies and Twins” platform (EUROCAT) provides the most recent data collection of OFCs in Europe, updated in April 2024: By pooling 40 registries, it reports an overall OFC prevalence of 1:767 newborns between 2007 and 2021 (including live, still births, and genetic anomalies) [16]. However, there are significant differences in the frequency of OFCs throughout the various European nations. Between 2007 and 2021, the northern countries have the highest prevalence (*e.g.,* Norway: 1:645 newborns), while the southern countries show the lowest numbers (*e.g.*, Portugal: 1:1,667). Swiss healthcare facilities contribute inconsistent documentation on OFC patients, with the only Swiss health care facilities participating in EUROCAT data collection being the hospitals in the canton of Vaud, reporting an OFC prevalence of 1:741 newborns. Even though this is a relatively high prevalence for Europe—more in line with the northern than southern European nations—it must be noted that in 2021 only 10% of Swiss live births (LB) occurred in canton Vaud [17], so its data cannot be extrapolated to the entire nation.

This study aims to report descriptive epidemiologic data from 2007 to 2021 on the actual numbers, prevalence, and trends of live cleft births in Switzerland. These findings are innovative and highly significant for clinical practice, as they may enhance our understanding of the etiology of OFCs, foster new research platforms, and the exploration of novel strategies for the best possible patient care in an effort to optimize treatment.

## Methods

### Data collection

All medical conditions are codified based on the “International Statistical Classification of Diseases and Related Health Problems” Version 10 (ICD-10) [18]. All Swiss medical institutions are committed to use this classification system as required by the Swiss Federal Law (Federal Statistics Act of 9 October 1992 (FStatA); SR 431.01 and SR 431.012.1) [19]. Data are then collected by the Federal Statistical Office (FSO) in a centralized register. They included all registered LB cases with OFCs as described by the ICD-10 (Table S1) between the years 1998 and 2021 for every canton in Switzerland. For each year and every Swiss canton, the number of OFC cases by ICD-10 code and their sex, as well as the total number of LB were available. A more detailed data pool, such as associated genetic anomalies, was not provided by the FSO to protect patient’s anonymity, given the low number of cases recorded. Since the data were collected from the FSO, which provided the cases without any possibility of specific subject identification, this study did not require ethic board approval.

### Reliability of the data

As data collection was not mandatory in all cantons in the late nineties, we had to examine the reliability of our data. Indeed, the participation rate was rather low and not representative for the entire Swiss population between 1998 and 2006 (Table S2). Today, every health facility in Switzerland is committed by law to report the diagnosis, treatment and costs for each patient. The years of obvious under-reporting of OFC cases in Switzerland (1998–2006, Table S2) were excluded from our analysis to avoid introducing a bias in the results. Therefore, numbers and prevalences of OFC cases reported in the text always refer to the years 2007–2021.

### Data analysis

Data were inserted in a spreadsheet (Microsoft Excel Version 16.24; 2019; Microsoft, Redmond, WA, USA). Descriptive and bivariate analysis were performed using the software STATA 17^(R)^. Prevalence is reported *per* 10,000 live births (LB). Prevalence = (number of cases / number of LB) × 10,000.

## Results

### Proportion of cleft types in Switzerland between 2007 and 2021

Between 2007 and 2021, there were a total of 1,252,155 live births (LB) registered in Switzerland (Table 1, yellow line). Of these, 1,567 (0.13%) newborns presented an OFC (on average 104.5 cases/year; 95% CI: 100.43–108.57).

**Table 1:**
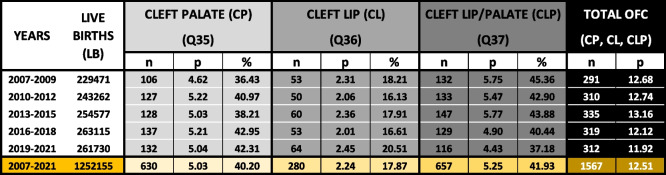
Epidemiologic profile of OFC cases in Switzerland between 2007 and 2021

Number of live births (LB), number of cases (n), prevalence (p)/10,000 LB, and the proportions in % (n cases of a cleft type/n cases in total) for cleft palate (CP – Q35), cleft lip (CL – Q36), and cleft lip with cleft palate (CLP – Q37) and total OFC (sum of CP, CL, and CLP) in Switzerland. Because of low numbers, data derived from three consecutive years were pooled. The bottom line, highlighted in yellow, shows the average data between 2007 and 2021, for which reliable reporting of the OFC cases was achieved. (OFC: orofacial clefts)

The distribution of the main three OFC phenotypes (Table S1) according to ICD-10 [18] between 2007 and 2021 was: Q35—cleft palate (CP): 40.2% (*n* = 630; on average 42.0/year; 95% CI: 39.1–44.9), Q36—cleft lip (CL): 17.9% (*n* = 280; on average 18.7/year; 95% CI: 16.24–21.16; 89.8% were unilateral belonging to subtype Q36.9), and Q37—cleft lip with cleft palate (CLP): 41.9% (*n* = 657, on average 43.8/year; 95% CI: 40.96–46.64; 74.2% were unilateral belonging to subtypes Q37.1, Q37.3, Q37.5, and Q37.9) (Table 1 and Table S2).

**Fig. 1:**
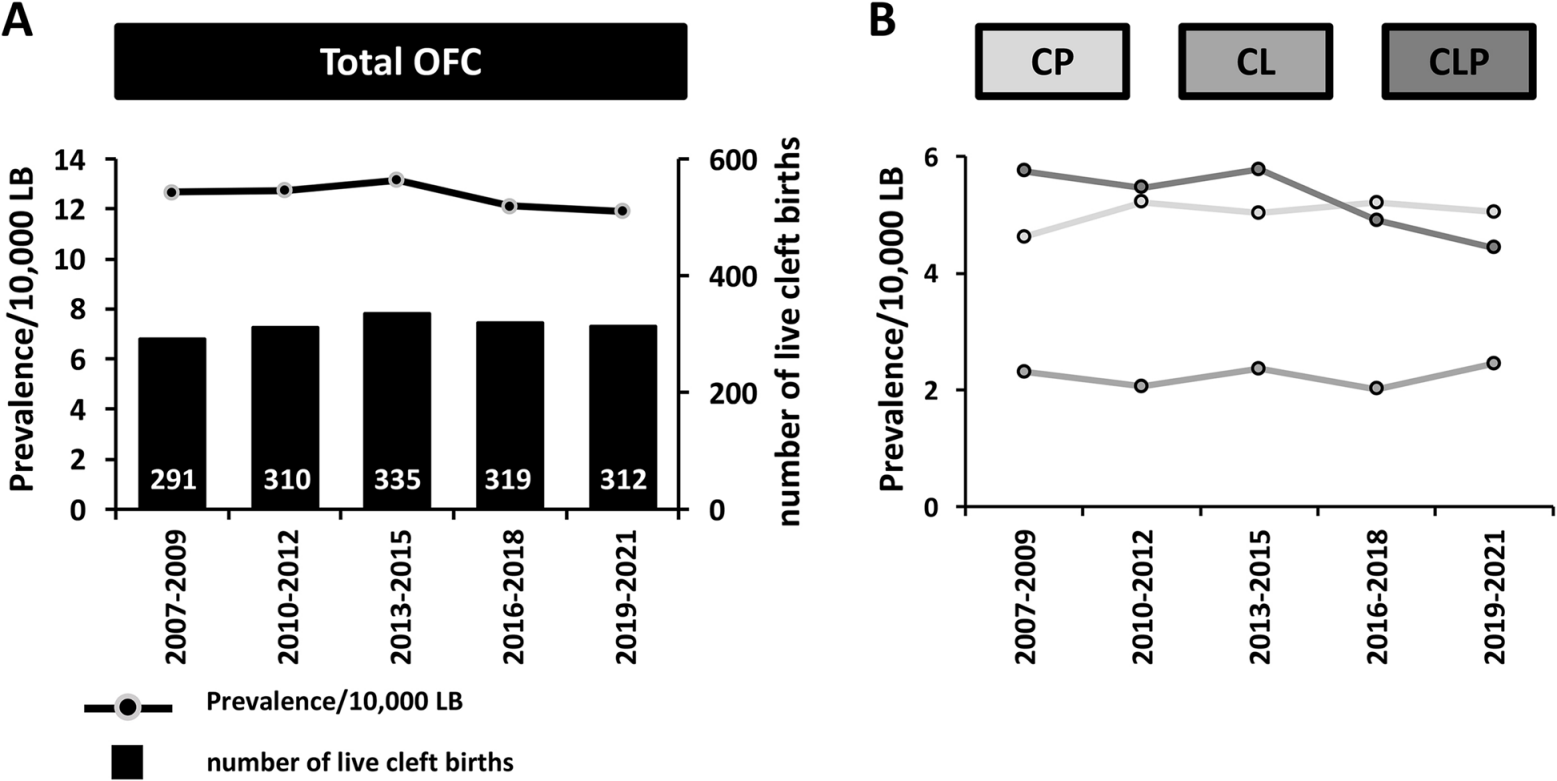
OFC numbers and prevalence per 10,000 live births in Switzerland between 2007 and 2021. **A** Number of live cleft births (bars, right axis) and total OFC prevalence in Switzerland between 2007 and 2021 (line, left axis). Because of low numbers, data derived from three consecutive years were pooled. The numbers indicate the actual total OFC cases reported within the indicated periods. **B** Prevalence of cleft palate (CP), cleft lip (CL), and cleft lip with cleft palate (CLP) per 10,000 LB in Switzerland between the different time periods. (OFC: orofacial clefts; LB: live births.)

The trend of OFC prevalence is shown in Fig. 1A. At the national Swiss level, the prevalence per 10,000 LB with OFC between 2007 and 2021 was 12.5 (95% CI: 12.03–12.97; corresponding to 1:799 newborns), with a relatively stable trend (0.94-fold change from the time periods 2007–2009 to 2019–2021). Figure 1B displays the individual prevalence per 10,000 LB of CP, CL, and CLP between 2007 and 2021, which were 5.03 (95% CI: 4.74–5.32; corresponding to 1:1,988 newborns), 2.24 (95% CI: 1.95–2.53; corresponding to 1:4,464 newborns), and 5.25 (95% CI: 4.86–5.64; corresponding to 1:1,905 newborns), respectively. While there seemed to be a relatively stable trend for CP and CL between 2007–2009 and 2019–2021 (CP: × 1.09; CL: × 1.06), the prevalence of CLP decreased (× 0.77) in the same time interval. These numbers are mirrored in the proportional distribution of the three distinct OFC phenotypes between 2007 and 2021: while CP and CL increased from 36.4% to 42.3% and 18.2% to 20.5%, CLP declined from 45.4% to 37.2% (Table 1).

### Sex distribution

**Fig. 2:**
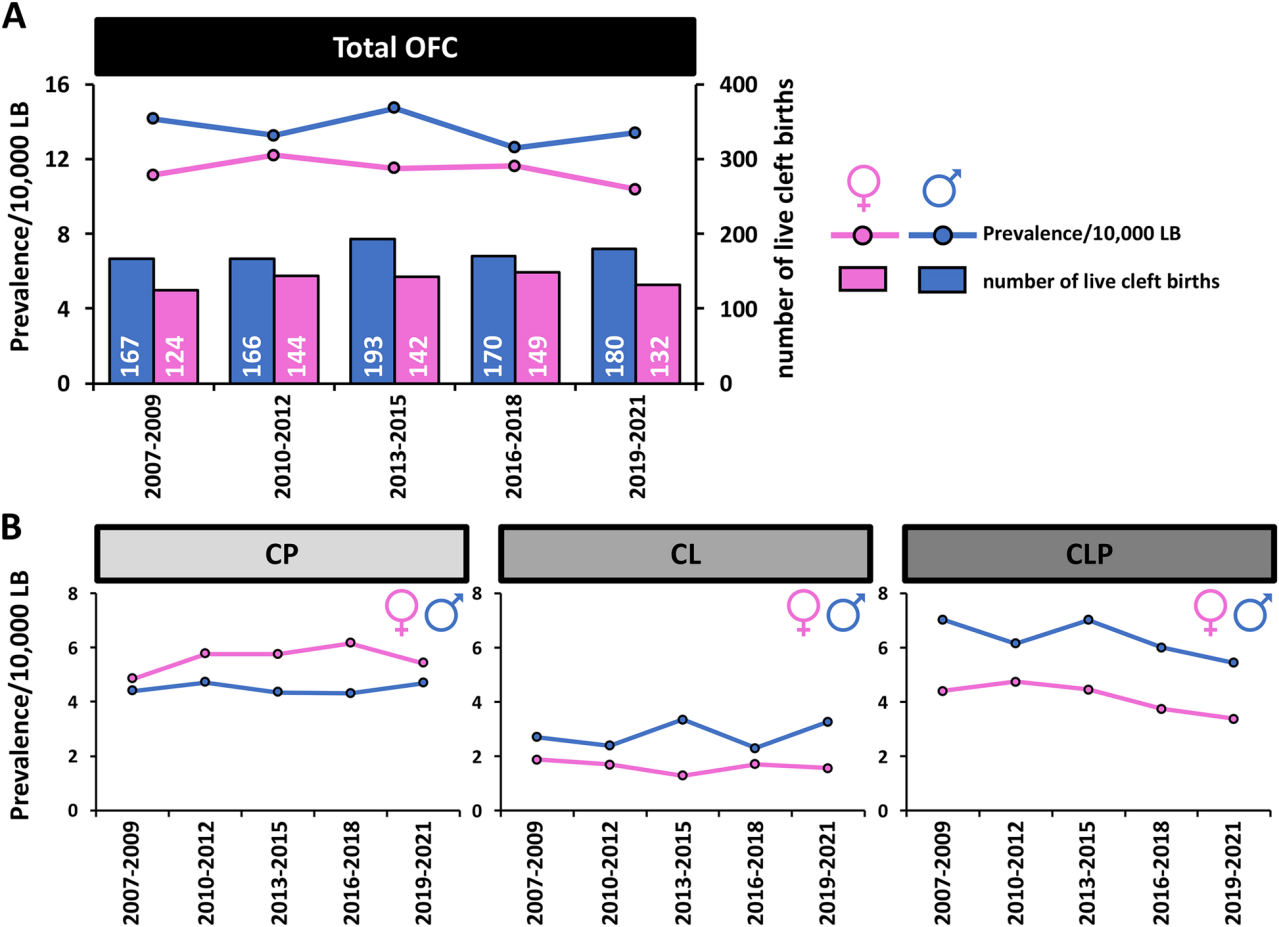
Cleft types and sex distribution. **A** Number of live cleft births (bars, right axis) and total OFC prevalence (lines, left axis), divided into males (blue) and females (pink) in Switzerland between 2007 and 2021. The numbers indicate the total OFC cases reported within the indicated periods. **B** Prevalence of cleft palate (CP), cleft lip (CL), and cleft lip with cleft palate (CLP) according to sex in Switzerland between 2007 and 2021 per 10,000 LB. (OFC: orofacial clefts; LB: live births)

The sex distribution for the total OFC cases, as well as for the different cleft types, is reported in Table 2. Considering newborns with OFCs between 2007 and 2021, males are more likely to develop OFCs than females. Taking into account all OFC, there were 876 males (on average 58.4/year; 95% CI: 54.53–62.27) and only 691 females (on average 46.1/year; 95% CI: 43.36–48.84). Total cleft prevalence was 13.6 in males (95% CI: 12.72–14.48; corresponding to 1:735 newborns) and 11.35 in females (95% CI: 10.69–12.01; corresponding to 1:881 newborns). Hence, approximately 20% more males were affected by OFCs than females (male/female ratio (M/F): 1.2), a number that remained relatively stable during the last 15 years (Table 2 and Fig. 2A). CL and CLP were more prevalent in males compared to females between 2007 and 2021, with M/F ratios of 1.73 and 1.53, respectively (Table 2). In contrast, CP was more prevalent in females (M/F: 0.80) (Table 2). Additionally, while there was a slightly decreasing trend for CLP in both sexes during the time periods 2007–2009 to 2019–2021 (× 0.77 decrease for males and females), CP and CL displayed a relatively stable trend for both sexes within the same time frame (Table 2 and Fig. 2B).

**Table 2:**
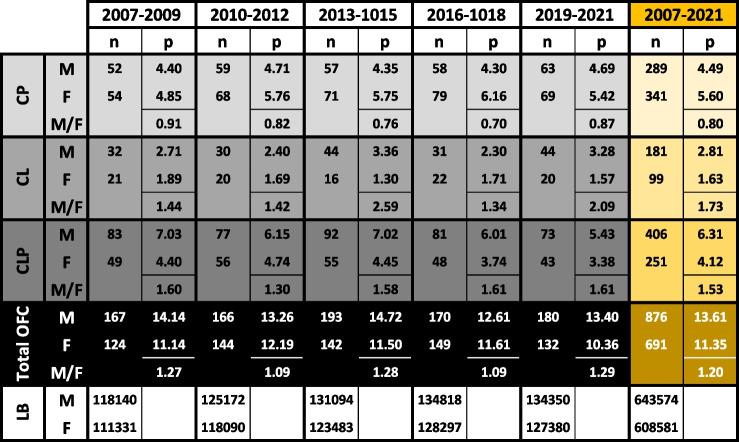
Sex distribution of OFC cases in Switzerland

Sex distribution of cleft palate (CP), cleft lip (CL), cleft lip with cleft palate (CLP), and total OFC cases is shown. The number of cases (n) and prevalence (p)/10,000 LB are indicated for each cleft type within a time interval. M/F shows the relative sex ratio (normalized to male and female LB, respectively). The last lines (highlighted in yellow) report the collective data between 2007 and 2021. M: male; F: female. (OFC: orofacial clefts)

### Regional distribution

**Fig. 3:**
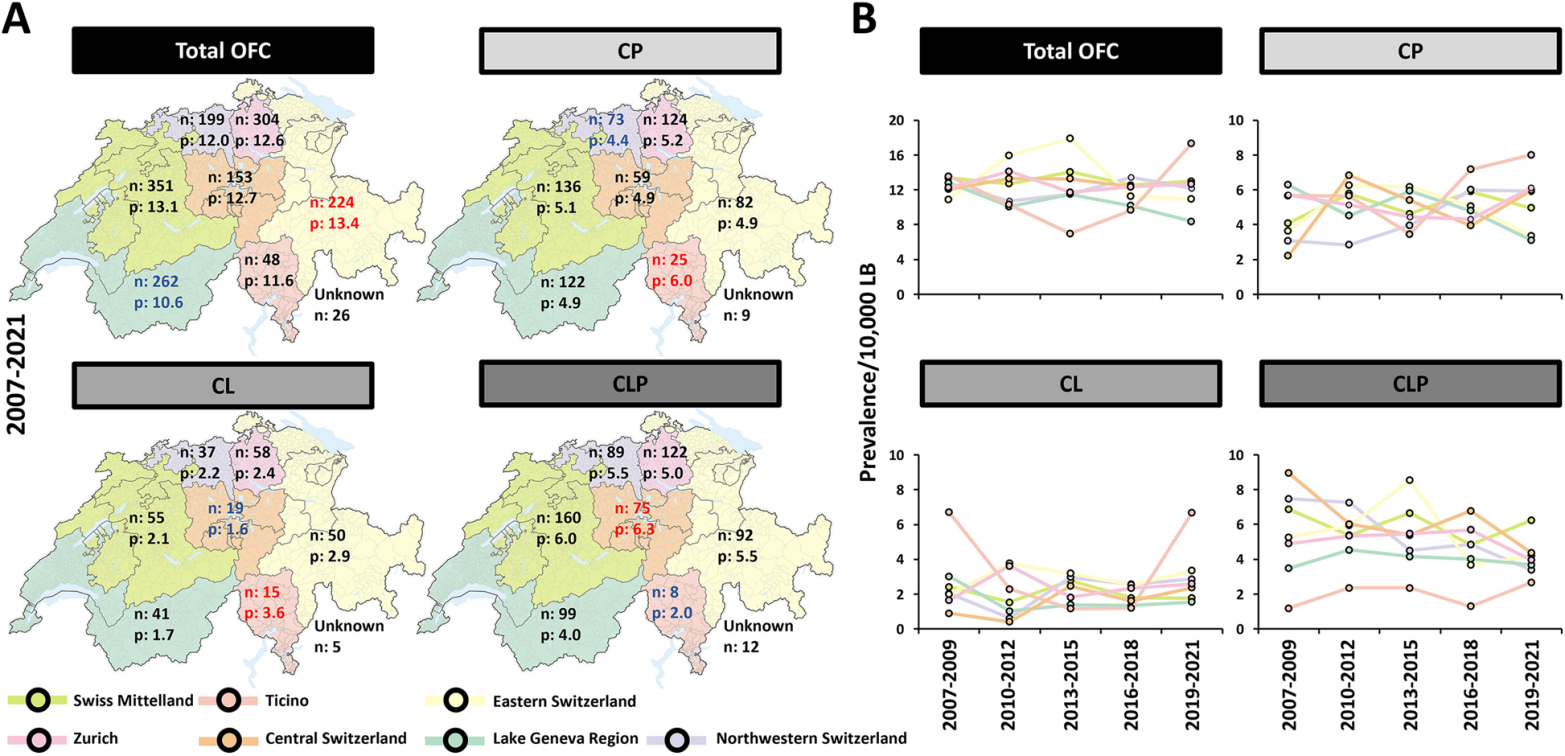
Distribution of OFC cases in Switzerland. **A** Color-coded geographical maps indicating the seven official regions of Switzerland: Swiss Mittelland (light green), Zurich (pink), Ticino (red), Central Switzerland (orange), Eastern Switzerland (yellow), Lake Geneva Region (dark green), and Northwestern Switzerland (purple). The number of total cases of cleft palate (CP), cleft lip (CL), cleft lip with cleft palate (CLP), and total OFC cases is shown for the years 2007–2021 as well as their prevalence for each of the regions. Red font: region with the highest prevalence; blue font: region with the lowest prevalence. Shown are also the number of newborns with unknown Swiss home region. The map of Switzerland was taken from https://de.wikipedia.org/wiki/Grossregion_(Schweiz)# under the creative commons CC BY-SA 3.0. **B** Prevalence of cleft palate (CP), cleft lip (CL) or cleft lip with cleft palate (CLP), and total OFC cases between 2007 and 2021 for the seven Swiss regions per 10,000 LB. (n: number; p: prevalence/10,000 LB; OFC: orofacial cleft; LB: live births)

Finally, we determined the number of CP, CL, and CLP cases and their prevalence within the seven official Swiss regions (*i.e*., Swiss Mittelland, Northwestern Switzerland, Eastern Switzerland, Lake Geneva Region, Ticino, Central Switzerland, and Zurich) (Table 3). The regional total OFC prevalence per 10,000 LB between 2007 and 2021 varied considerably (Fig. 3), with Eastern Switzerland (13.36; 95% CI: 11.05–15.67; corresponding to 1:749 newborns) and Lake Geneva Region (10.56; 95% CI: 8.83–12.29; corresponding to 1:947 newborns) being the most (red font) and least affected areas (blue font), respectively. Similarly, the three OFC subtypes displayed uneven geographical distributions within Switzerland (Fig. 3A): Ticino had the highest prevalence per 10,000 LB of both CP (6; 95% CI: 3.26–8.74; corresponding to 1:1,667 newborns) and CL (3.59; 95% CI: 1.05–6.13; corresponding to 1:2,786 newborns), while CLP was most frequently observed in Central Switzerland (6.27; 95% CI: 5.19–7.35; corresponding to 1:1,595 newborns). In contrast, CP, CL, and CLP showed the lowest prevalence in Northwestern Switzerland (4.37; 95% CI: 3.39–5.35; corresponding to 1:2,288), Central Switzerland (1.55; 95% CI: 0.77–2.33; corresponding to 1:6,452), and Ticino (1.97; 95% CI: 0.61–3.33; corresponding to 1:5,076), respectively (Fig. 3A). These observations were independent of the sex of the newborns (Table 3).

**Table 3:**
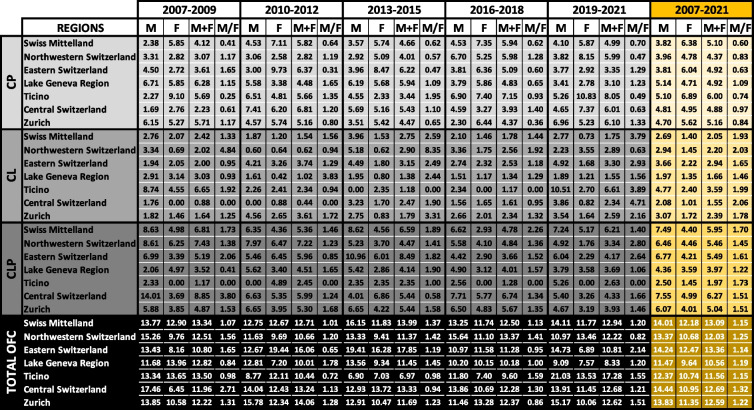
Sex distribution of OFC within the various Swiss regions

The seven official regions of Switzerland were analyzed for the prevalence (p)/10,000 LB of cleft palate (CP), cleft lip (CL), cleft lip with cleft palate (CLP), and total OFC cases in males (M) and females (F) and total newborns (M+F). M/F shows the normalized ratio between males and females. The last columns (highlighted in yellow) specifically report the averaged numbers between 2007 and 2021. (OFC: orofacial clefts; LB: live births)

Figure 3B shows the regional trends for total OFC, CP, CL, and CLP from 2007–2009 to 2019–2021. Fluctuating patterns were observed in the intra-regional prevalence of total OFC cases and for the various cleft subtypes throughout all seven Swiss regions between 2007–2009 and 2019–2021. The most notable variation amongst the regions was Ticino’s much lower prevalence of CLP cases when compared to the other areas.

In terms of sex distribution, all Swiss regions had a higher prevalence of male newborns with total OFCs, CL, and CLP (Table 3). In contrast, CP was more common in female newborns in all regions, except the Lake Geneva Region.

## Discussion

To the best of the author’s knowledge, this is the first descriptive report on the epidemiology of OFC in Switzerland. Although we obtained data from the FSO starting in 1998, there was a significant underreporting of the OFC cases between 1998–2006 (Table S2). Therefore, to prevent biasing the results, only data from 2007 was analyzed.

Between 2007 and 2021, Switzerland recorded a total of 1,567 live cleft births, corresponding to 12.51 cases per 10,000 LB, or 1:799 newborns. Compared to Switzerland’s bordering nations, this overall OFC frequency is similar to data reported by EUROCAT for Austria (1:833 newborns) and France (1:870 newborns), higher than for Italy (1:1000 newborns), but lower than Germany (1:606 newborns) [16]. These comparisons should be interpreted with caution, as none of these countries offered data from national OFC registries but from regional registries. The prevalence of CP, CL, and CLP in Switzerland was determined to be 5.03, 2.24, and 5.25 per 10,000 LB, respectively, between 2007 and 2021 (Table 1). Switzerland had more newborns affected by CP and CLP but fewer cases of CL than the recently reported global prevalence of 3.3 (CP), 3 (CL), and 4.5 (CLP) per 10,000 LB [20]. CL (18%) is by far the least prevalent cleft subtype, with CP (40.2%) and CLP (41.9%) representing the wide majority (Table 1). However, the distributions of the various phenotypes are in line with recent epidemiologic studies from bordering countries (Germany, Lower Saxony [21], and Italy, Emilia-Romagna and Tuscany [22]).

The sex distribution of OFCs in Switzerland differed significantly, according to our population-based data (Table 2 and Fig. 2). Overall OFC cases, as well as CL and CLP, are more common in males than in females, which is consistent with findings from earlier global studies [7, 23], but females had a noticeably higher prevalence of CP. This could be explained by the slightly delayed development of the secondary palate in female fetuses compared to male fetuses [24], with the palatal shelves in females remaining separated for an additional week compared to males [25, 26]. This delayed secondary palate closure may provide a longer window of exposure to teratogens, which could interfere with normal palatogenesis.

Switzerland is a small country, with approximately 8.8 million inhabitants living on 41,285 km^2^ in 2022. The Swiss population represents a varied range of social and ethnic groups. According to the FSO (www.atlas.bfs.admin.ch/maps/13/de/17505_90_89_70/27127.html), the cantons of Geneva, Vaud (both in the Lake Geneva Region), Basel (Northwestern Switzerland), and Ticino have among the highest percentages of permanent foreign populations, ranging from 28.1% (Ticino) to 64.4% (Geneva) in the year 2022. Considering that OFCs may have a genetic component, the number of people from countries with different genetic backgrounds may have an impact on the prevalence of OFCs. However, as immigration is determined by a variety of factors (*e.g.*, job opportunities, family), it is clearly oversimplified to assume, for instance, that Italians, who seem to have a lower OFC prevalence than Swiss, migrate primarily to the Italian-speaking cantons of Ticino and Grisons, lowering OFC numbers in these regions. Based on these thoughts, epidemiologic research on OFC in Switzerland is challenging to interpret, and we were unable to identify a consistent association between geographic region and OFC prevalence in Switzerland (Table 3 and Fig. 3). Notably, Swiss regions along the borders with neighboring countries often had the most extreme (highest and lowest) prevalence of total OFC cases and cleft subtypes. It is unclear and speculative if these areas have a high level of ethnic diversity, distinct behaviors, or environmental exposures that could explain their comparatively higher OFC prevalence, or if this can be explained by a low birth rate artificially inflating the prevalence. It should be noted that the OFC figures provided for Ticino are low; therefore, even a small number of unreported cases could drastically change the regional prevalence.

Of note, while the average mother’s birth age in Switzerland increased from 30.7 ± 0.41 years in 2007 to 32.3 ± 0.55 years in 2021, the prevalence of OFC remained stable, suggesting either that the maternal age difference is too small or that maternal age may not be a significant risk factor for OFC.

OFC can be diagnosed by prenatal 2D transabdominal ultrasonography at around week 13 of pregnancy. However, CL in the low-risk population is only diagnosed by routine ultrasonic scans at week 20 of gestation in 16% to 93% of the cases, which indicates a significant rate of missed diagnosis [27]. A recent monocentric retrospective study in Switzerland reported variable diagnosing rates of 58.2%, 91.3%, and 2% for CL, CLP, and CP, respectively [28]. The development of 3D- or 4D-ultrasound techniques can improve the precision of OFC diagnosis [29], although the reliable identification of CP is still difficult [27]. Prenatal OFC detection mandates parental guidance and education by a cleft expert team, allowing the parents to make an informed decision about the pregnancy [30]. It could be assumed that a higher rate of abortions would result from prenatal OFC identification. However, to date, the consequences of the improved prenatal discovery of OFC on pregnancy termination are scarcely reported, with conflicting results [31–33].

The present study has several limitations. Neither the abortions motivated by the prenatal discovery of OFCs nor the presence of syndromes or facial clefts (Tessier clefts) were available in our data sets. Abortions are reported to occur more frequently in syndromic OFC cases [34–36]. Additionally, some cleft phenotypes (*e.g*., cleft uvula and submucosal clefts) are challenging to identify by visual inspection perinatally; therefore, the overall number of OFC cases is likely higher than what is reported in this study. There is no information available on the possible familial history of OFC, which would be extremely pertinent given the risk of cleft recurrence in first-degree relatives [37]. Furthermore, we have no data on parents’ socio-ethnic background and habits (*e.g*., smoking), which would allow for more correlative studies. Lastly, it should be noted that the cleft classification method used is also crucial for studying the epidemiology of OFCs [38]. It should be precise without being overly intricate or impractical. Unfortunately, there is currently no internationally recognized classification system [10, 39]. Our analysis was derived from the ICD-10 classification system, which is neither extremely specific nor exclusive in phenotypic descriptions for categorizing OFCs. As a result, the completeness of the clefts, cleft laterality, and alveolar status are not indicated by the ICD-10 codes [40]. It appears clear that a universally used classification system for clefts is essential for better comprehension and transfer of scientific data.

## Conclusions

We present the first descriptive epidemiologic profiles for OFCs in Switzerland. While this study provides new data helping to understand cleft epidemiology, there is a clear need for a more precise and improved reporting system. This has been acknowledged among the OFC experts in Switzerland, and consequently, the Swiss Cleft Registry (https://swisscleftregistry.org) was launched nationwide in 2011. This should enhance comprehension and facilitate the identification of potential risk factors involved in OFC etiology. The Swiss Registry also seeks to offer an interdisciplinary platform to support collaborative cleft research endeavors in Switzerland, which will hopefully benefit patients with OFCs and their families. However, to be successful and relevant, registries need the full commitment of all involved health care practitioners, an accurate diagnosis, and consistent reporting.

## Supplementary Information

Supplementary Material 1.

## Data Availability

The datasets generated and/or analyzed during the current study are available from the corresponding author on reasonable request.

## Abbreviations

CL: Cleft lip
CLP: Cleft lip with cleft palate
CP: Cleft palate
EUROCAT: European registration of congenital anomalies and twins
FSO: Federal Statistical Office
OFC: Orofacial clefts
LB: Live births
OFC: Orofacial clefts

## References

[1] Wehby GL, Cassell CH. The impact of orofacial clefts on quality of life and healthcare use and costs. Oral Dis. 2010;16(1):3–10. 19656316 10.1111/j.1601-0825.2009.01588.x PMC2905869

[2] Wang D, Zhang B, Zhang Q, Wu Y. Global, regional and national burden of orofacial clefts from 1990 to 2019: an analysis of the Global Burden of Disease Study 2019. Ann Med. 2023;55(1):2215540. 37232757 10.1080/07853890.2023.2215540 PMC10228319

[3] Rahimov F, Jugessur A, Murray JC. Genetics of nonsyndromic orofacial clefts. Cleft Palate Craniofac J. 2012;49(1):73–91. 21545302 10.1597/10-178 PMC3437188

[4] Nasreddine G, El Hajj J, Ghassibe-Sabbagh M. Orofacial clefts embryology, classification, epidemiology, and genetics. Mutat Res Rev Mutat Res. 2021;787:108373. 34083042 10.1016/j.mrrev.2021.108373

[5] Leslie EJ, Carlson JC, Shaffer JR, Feingold E, Wehby G, Laurie CA, Jain D, Laurie CC, Doheny KF, McHenry T, et al. A multi-ethnic genome-wide association study identifies novel loci for non-syndromic cleft lip with or without cleft palate on 2p24.2, 17q23 and 19q13. Hum Mol Genet. 2016;25(13):2862–72. 27033726 10.1093/hmg/ddw104 PMC5181632

[6] Schutte BC, Murray JC. The many faces and factors of orofacial clefts. Hum Mol Genet. 1999;8(10):1853–9. 10469837 10.1093/hmg/8.10.1853

[7] Dixon MJ, Marazita ML, Beaty TH, Murray JC. Cleft lip and palate: understanding genetic and environmental influences. Nat Rev Genet. 2011;12(3):167–78. 21331089 10.1038/nrg2933 PMC3086810

[8] Stanier P, Moore GE. Genetics of cleft lip and palate: syndromic genes contribute to the incidence of non-syndromic clefts. Hum Mol Genet. 2004;13 Spec No 1:R73-81. 14722155 10.1093/hmg/ddh052

[9] Luijsterburg AJ, Rozendaal AM, Vermeij-Keers C. Classifying common oral clefts: a new approach after descriptive registration. Cleft Palate Craniofac J. 2014;51(4):381–91. 23432103 10.1597/1569-51.4.493

[10] Watkins SE, Meyer RE, Strauss RP, Aylsworth AS. Classification, epidemiology, and genetics of orofacial clefts. Clin Plast Surg. 2014;41(2):149–63. 24607185 10.1016/j.cps.2013.12.003

[11] McBride WA, McIntyre GT, Carroll K, Mossey PA. Subphenotyping and Classification of Orofacial Clefts: Need for Orofacial Cleft Subphenotyping Calls for Revised Classification. Cleft Palate Craniofac J. 2016;53(5):539–49. 26171570 10.1597/15-029

[12] Ray HJ, Niswander L. Mechanisms of tissue fusion during development. Development. 2012;139(10):1701–11. 22510983 10.1242/dev.068338 PMC3328173

[13] Burg ML, Chai Y, Yao CA, Magee W 3rd, Figueiredo JC. Epidemiology, Etiology, and Treatment of Isolated Cleft Palate. Front Physiol. 2016;7:67. 26973535 10.3389/fphys.2016.00067 PMC4771933

[14] Kohli SS, Kohli VS. A comprehensive review of the genetic basis of cleft lip and palate. J Oral Maxillofac Pathol. 2012;16(1):64–72. 22438645 10.4103/0973-029X.92976 PMC3303526

[15] Yankee TN, Oh S, Winchester EW, Wilderman A, Robinson K, Gordon T, Rosenfeld JA, VanOudenhove J, Scott DA, Leslie EJ, et al. Integrative analysis of transcriptome dynamics during human craniofacial development identifies candidate disease genes. Nat Commun. 2023;14(1):4623. 37532691 10.1038/s41467-023-40363-1 PMC10397224

[16] EUROCAT - European network of population-based registries for the epidemiological surveillance of congenital anomalies. https://eu-rd-platform.jrc.ec.europa.eu/eurocat_en.

[17] Lebendgeburten nach Kanton und Staatsangehörigkeitskategorie der Mütter, 1970–2022. https://www.bfs.admin.ch/bfs/en/home/statistics/population/births-deaths/births.assetdetail.25565518.html.

[18] International Classification of Diseases, Tenth Revision (ICD-10). www.cdc.gov/nchs/icd/icd-10/?CDC_AAref_Val=, https://www.cdc.gov/nchs/icd/icd10.htm.

[19] Swiss Confederation: Federal Statistics Act (FStatA) of 9 October 1992 (Status as of 1 January 2024). https://www.fedlex.admin.ch/eli/cc/1993/2080_2080_2080/en.

[20] Salari N, Darvishi N, Heydari M, Bokaee S, Darvishi F, Mohammadi M. Global prevalence of cleft palate, cleft lip and cleft palate and lip: A comprehensive systematic review and meta-analysis. J Stomatol Oral Maxillofac Surg. 2022;123(2):110–20. 34033944 10.1016/j.jormas.2021.05.008

[21] Kauffmann P, Quat A, Schmunke B, Kolle J, Wolfer S, Stepniewski A, Meyer-Marcotty P, Schliephake H. Epidemiological and clinical evaluation of patients with a cleft in lower saxony Germany: a mono-center analysis. Clin Oral Investig. 2023;27(9):5661–70. 10.1007/s00784-023-05187-9 PMC10492882 37542681

[22] Impellizzeri A, Giannantoni I, Polimeni A, Barbato E, Galluccio G. Epidemiological characteristic of Orofacial clefts and its associated congenital anomalies: retrospective study. BMC Oral Health. 2019;19(1):290. 31870360 10.1186/s12903-019-0980-5 PMC6929424

[23] Mossey PA, Modell B. Epidemiology of oral clefts 2012: an international perspective. Front Oral Biol. 2012;16:1–18. 22759666 10.1159/000337464

[24] Burdi AR, Faist K. Morphogenesis of the palate in normal human embryos with special emphasis on mechanisms involved. Am J Anat. 1967;120:149–60.

[25] Burdi AR, Silvey RG. Sexual differences in closure of the human palatal shelves. Cleft Palate J. 1969;6:1–7. 5251435

[26] Merritt L. Part 1. Understanding the embryology and genetics of cleft lip and palate. Adv Neonatal Care. 2005;5(2):64–71. 15806447 10.1016/j.adnc.2004.12.006

[27] Maarse W, Berge SJ, Pistorius L, van Barneveld T, Kon M, Breugem C. Mink van der Molen AB: Diagnostic accuracy of transabdominal ultrasound in detecting prenatal cleft lip and palate: a systematic review. Ultrasound Obstet Gynecol. 2010;35(4):495–502. 20235140 10.1002/uog.7472

[28] Guichoud Y, El Ezzi O, de Buys Roessingh A. Cleft Lip and Palate Antenatal Diagnosis: A Swiss University Center Performance Analysis. Diagnostics (Basel). 2023;13(15):2479. 37568842 10.3390/diagnostics13152479 PMC10416856

[29] Rotten D, Levaillant JM. Two- and three-dimensional sonographic assessment of the fetal face. 2. Analysis of cleft lip, alveolus and palate. Ultrasound Obstet Gynecol. 2004;24(4):402–11. 15343594 10.1002/uog.1718

[30] Berggren H, Hansson E, Uvemark A, Svensson H, Becker M. Prenatal compared with postnatal cleft diagnosis: what do the parents think? J Plast Surg Hand Surg. 2012;46(3–4):235–41. 22909240 10.3109/2000656X.2012.698416

[31] Nusbaum R, Grubs RE, Losee JE, Weidman C, Ford MD, Marazita ML. A qualitative description of receiving a diagnosis of clefting in the prenatal or postnatal period. J Genet Couns. 2008;17(4):336–50. 18481163 10.1007/s10897-008-9152-5

[32] Han HH, Choi EJ, Kim JM, Shin JC, Rhie JW. The Importance of Multidisciplinary Management during Prenatal Care for Cleft Lip and Palate. Arch Plast Surg. 2016;43(2):153–9. 27019808 10.5999/aps.2016.43.2.153 PMC4807170

[33] Emodi O, Capucha T, Shilo D, Ohayon C, Ginini JG, Ginsberg Y, Aizenbud D, Rachmiel A. Trends in cleft palate incidence in the era of obstetric sonography and early detection. J Matern Fetal Neonatal Med. 2022;35(25):9350–5. 35129039 10.1080/14767058.2022.2032635

[34] Li Z, Ren A, Liu J, Zhang L, Ye R, Li S, Li Z. High prevalence of orofacial clefts in Shanxi Province in northern China, 2003–2004. Am J Med Genet A. 2008;146A(20):2637–43. 18798320 10.1002/ajmg.a.32492

[35] Russell KA, Allen VM, MacDonald ME, Smith K, Dodds L. A population-based evaluation of antenatal diagnosis of orofacial clefts. Cleft Palate Craniofac J. 2008;45(2):148–53. 18333649 10.1597/06-202.1

[36] Vallino-Napoli LD, Riley MM, Halliday JL. An epidemiologic study of orofacial clefts with other birth defects in Victoria. Australia Cleft Palate Craniofac J. 2006;43(5):571–6. 16986995 10.1597/05-123

[37] Sivertsen A, Wilcox AJ, Skjaerven R, Vindenes HA, Abyholm F, Harville E, Lie RT. Familial risk of oral clefts by morphological type and severity: population based cohort study of first degree relatives. BMJ. 2008;336(7641):432–4. 18250102 10.1136/bmj.39458.563611.AE PMC2249683

[38] Houkes R, Smit J, Mossey P, Don Griot P, Persson M, Neville A, Ongkosuwito E, Sitzman T, Breugem C. Classification Systems of Cleft Lip, Alveolus and Palate: Results of an International Survey. Cleft Palate Craniofac J. 2023;60(2):189–96. 34812658 10.1177/10556656211057368 PMC9843539

[39] Wang KH, Heike CL, Clarkson MD, Mejino JL, Brinkley JF, Tse RW, Birgfeld CB, Fitzsimons DA, Cox TC. Evaluation and integration of disparate classification systems for clefts of the lip. Front Physiol. 2014;5:163. 24860508 10.3389/fphys.2014.00163 PMC4030199

[40] Aylsworth AS, Allori AC, Pimenta LA, Marcus JR, Harmsen KG, Watkins SE, Ramsey BL, Strauss RP, Meyer RE. Issues involved in the phenotypic classification of orofacial clefts ascertained through a state birth defects registry for the North Carolina Cleft Outcomes Study. Birth Defects Res A Clin Mol Teratol. 2015;103(11):899–903. 26251069 10.1002/bdra.23415

