# Supplementary material for: Epidemiologic trends of cleft lip and/or palate in Switzerland
Source: BMC Oral Health. 2025 Jan 22;25:114. doi: 10.1186/s12903-025-05500-w (PMC11756186; doi:10.1186/s12903-025-05500-w)
Supplement: Supplementary file 1 — Supplementary Material 1. [file 12903_2025_5500_MOESM1_ESM.docx]

**Epidemiologic trends of cleft lip and/or palate in Switzerland**

Joël Beyeler^1,2*^, Anic Lauener ^1*^, Christos Katsaros^1,3^, Giorgio C. La Scala^4#^, and Martin Degen^3#^

^1^Department of Orthodontics and Dentofacial Orthopedics, University of Bern, Bern, Switzerland

^2^Department of Restorative, Preventive and Pediatric Dentistry, School of Dental Medicine, University of Bern, Bern, Switzerland.

^3^Laboratory for Oral Molecular Biology, Department of Orthodontics and Dentofacial Orthopedics, University of Bern, Bern, Switzerland.

^4^Pediatric Plastic Surgery, Division of Pediatric Surgery, Department of Pediatrics, University Hospital of Geneva, Geneva, Switzerland

^*^ Equal first authors

# Shared last authorship

Correspondence: Martin Degen

Freiburgstrasse 3

3010 Bern, Switzerland

martin.degen@unibe.ch

**Supplementary Tables**

**Table S1 – ICD-10-coding system for orofacial clefts**

Definition of the various orofacial cleft subtypes and their unique and identifying ICD-10 codes.

**Table S2 – Under-reporting of OFC cases in Switzerland between 1998 and 2006**

Number of live births (LB), number of cases (n), prevalence (p)/10,000 LB, and the proportions in % (n cases of a cleft type/n cases in total) for cleft palate (CP – Q35), cleft lip (CL – Q36), and cleft lip and cleft palate (CLP – Q37) and total OFC (sum of CP, CL, and CLP) in Switzerland. Because of the low numbers, data derived from three consecutive years were pooled. The bottom line, highlighted in yellow, shows all data for the years 2007-2021, for which reliable reporting of the OFC cases was obtained. “Salmon colored” lines (years 1998-2006) indicate the years of obvious under-reporting. (OFC: orofacial clefts; LB: live births).
